# Multimodal healthcare system for human activity recognition using multiple features and advanced ensemble classifier

**DOI:** 10.1177/20552076261427061

**Published:** 2026-05-12

**Authors:** Fakhra Nazar, Yanfeng Wu, Hanan Aljuaid, Ahmad Jalal, Hui Liu

**Affiliations:** 1 372146 Guodian Nanjing Automation Co., Ltd, Nanjing, China; 2 Department of Computer Science, 53427 Air University, Islamabad, Pakistan; 3 Computer Sciences Department, College of Computer and Information Sciences, 112893 Princess Nourah bint Abdulrahman University (PNU), Riyadh, Saudi Arabia; 4 Department of Computer Science and Engineering, College of Informatics, Korea University, Seoul, South Korea; 5 School of Artificial Intelligence/School of Future Technology, Nanjing University of Information Science and Technology, Nanjing, China; 6 Cognitive Systems Lab, 9168 University of Bremen, Bremen, Germany

**Keywords:** Activity recognition, ensemble learning, healthcare, machine learning, sparse neural networks, wearable sensor

## Abstract

**Objective:**

Human activity recognition (HAR) is applicable in various areas as it helps in healthcare monitoring, exercise assessment, and monitoring of smart devices.

**Methods:**

To achieve high recognition, the study makes use of the synergistic integration of sophisticated signal processing and classification methods. First, we applied fourth-order median filtering and Hamming window processing to sensor signals, preserving activity-related changes while reducing excessive noise. Next, we extract several features, such as Shannon entropy, mel-frequency cepstral coefficients, spectral energy, spectral centroid, spectral flux, and dominant frequency, which enable us to gather information from both time and frequency domains. Subsequently, we adopt quadratic discriminant analysis to select the strongest features, facilitating easier identification of different classes. The final step involves training an ensemble of multi-layer perceptron (MLP), sparse MLP, and spatial-temporal MLP models, with all predictions made by each model combined through soft voting.

**Results:**

The proposed method demonstrates exceptional performance on three benchmark datasets, PAMAP2, Mobile Health, and Heterogeneity Human Activity Recognition, with accuracy values exceeding 95%.

**Conclusion:**

The results clearly illustrate the effectiveness and adaptability of the proposed HAR approach across various circumstances, regardless of who is performing the activity.

## Introduction

Human activity recognition (HAR) has gained significant importance across various fields, such as healthcare, ubiquitous computing, and smart environments, due to the increasing integration of smart devices and sensors into our daily lives.^1–4^ The rise of wearable and mobile technology has introduced challenges in real-time physical activity recognition and classification within pervasive computing. The growing popularity of HAR can be attributed to its versatility in application areas, including eldercare, physical activity monitoring, rehabilitation assistance, and enhancing human-computer interaction.^5–10^ HAR presents challenges because human movement is inherently complex, sensor placements can differ, and individual variations are abundant. The term multimodal used in this paper denotes the capability of collecting heterogeneous inertial sensing modalities (accelerometer, gyroscope, and magnetometer) that are based on different body locations (hand, chest, and ankle). All modalities provide different kinematic and orientation information, and their integration can give a complete representation of human motion to be used in healthcare monitoring.

Conventional approaches to HAR used specially designed features and then classified them with well-known classifiers. Nowadays, researchers are using more advanced techniques that take strong preprocessing, selective feature filtering, and combining methods to increase accuracy and adaptability.^11–13^ Various benchmark datasets have helped examine these methodologies in various kinds of experiments.^14,15^

We proposed a unique and efficient HAR system that includes preprocessing, engineering the right features, and another crucial part called classification. First, the Savitzky-Golay filter smoothed the initial sensor signal to cut out noise without distorting key elements. Thereafter, the signals were processed with a Hamming window to improve their quality before we could extract the features they contain. To represent human motion, we took six main features: Shannon entropy, mel-frequency cepstral coefficients (MFCCs), spectral energy, spectral centroid, spectral flux, and dominant frequency. To address redundancy and make our features more meaningful, we made use of quadratic discriminant analysis (QDA). Knowledge was built through a combination of three neural architectures, including a basic multi-layer perceptron (MLP), a spatial-temporal MLP (ST-MLP), and a sparse MLP. Soft voting performs the function of ensemble fusion, enhancing both accuracy and robustness. We carried out many experiments using the PAMAP2, Heterogeneity Activity Recognition Dataset, and Mobile Health (MHealth) datasets to check the effectiveness of the suggested approach.

Our HAR system has the following points as the major highlights:

- Savitzky-Golay filtering and Hamming windowing are used to increase the quality of the signal.
- All of the important features, such as entropy, cepstral, and frequency descriptions of human motion, are extracted.
- QDA helps select the best features so that the features are both relevant and separable.
- Three methods, MLP, ST-MLP, and sparse MLP, are used together in a single ensemble framework for strong activity recognition.

By experimenting on three popular datasets, the proposal was proven to be better than other methods in terms of accuracy and generalizability. The rest of the paper is organized this way: The “Related Work” section reviews previous research on HAR systems, the “Methodology” section covers our approach, which includes preprocessing and selecting features and techniques to classify data, the “Results” section looks at the experiments and how the proposed method performed and showed results, the “Discussion, Research Limitations, and Future Work” section includes discussions and points out limitations and possible future topics, and the “Conclusion” section closes the study.

## Related work

The rapid advancement of HAR in recent years can be attributed to the emergence of wearable sensing devices and the application of machine learning and deep learning algorithms. Numerous strategies for HAR have been proposed, which can be broadly categorized into two main types: traditional machine learning algorithms and deep learning approaches.

### Machine learning-based approaches

Earlier systems utilized human-engineered features such as mean, standard deviation, and signal entropy derived from accelerometer and gyroscope data. These systems implemented classification techniques like decision trees, k-nearest neighbors (k-NN), and support vector machines (SVMs).^16,17^ While these models showed promising results in laboratory settings, their lack of automation and the impact of sensor placement on performance led to challenges in real-world applications. Shoaib et al.^18^ highlighted these limitations in 2016, demonstrating that leveraging the locations of motion sensors on the wrist and in pockets significantly improved the identification of both repetitive and non-repetitive hand-oriented activities, particularly with optimal field window size selections. In 2018, Dong and Han introduced HARNet, which combined hand-crafted features with representations derived from convolutional neural networks (CNNs), achieving an accuracy improvement of 0.9 percent over SVM-based baselines.^19^ In 2020, Ehsan et al.^20^ evaluated decision trees, k-NN, logistic regression, SVM, and random forests using smartphone sensor data. The k-NN and SVM classifiers demonstrated the highest accuracy across various activities, including walking, navigating stairs, sitting, standing, and lying down. In 2021, Abidine and Fergani proposed a weighted LDA/SVM-KNN hybrid model that surpassed conventional classifiers in precision and F-score across a diverse range of HAR datasets following parameter optimization.^21^ The following year, Muhanad and Abdulah tested ten popular machine learning models on the M-HEALTH dataset, which included decision trees (DT), artificial neural networks (ANN), naïve Bayes (NB), k-NN, SVM, random forests (RF), and XGBoost. XGBoost emerged as the top-performing model, achieving approximately 0.99 F1 scores.^22^ These studies illustrate a gradual evolution in machine learning methodologies for HAR, moving from basic statistical models to more complex ensemble and hybrid machine learning algorithms. Despite their effectiveness in controlled laboratory environments, issues such as limitations, sensor variability, noise, and generalizability are significant challenges that will drive future advancements in this field.

### Deep learning-based approaches

Advancements in HAR have primarily resulted from deep learning techniques. CNNs are capable of extracting spatial features directly from the original sensor data, while Long Short-Term Memory (LSTM) networks, when used with Recurrent Neural Networks (RNNs), effectively capture sequential relationships in time-series signals.^23^ By integrating CNNs and LSTMs, hybrid models significantly enhance the detection and classification of movements, as they can recognize both the shape and temporal progression of actions.^24^

Sedaghati et al. introduced the IHARDS-CNN, a deep 1D CNN trained on various datasets, achieving nearly 100% accuracy without the need for result combination.^25^ Zhou et al. developed a DeepConv LSTM system capable of real-time HAR on small devices. This model utilizes minimal processing power to identify patterns across both spatial and temporal dimensions, making it well-suited for applications in healthcare and the Internet of Things (IoT).^26^ Sharma et al. proposed employing a CNN-LSTM approach on three datasets, MHealth, OPPORTUNITY, and HARTH, resulting in an accuracy as high as 99.07%. Their findings demonstrated that deep hybrid models can be effectively applied to a variety of sensors and activities.^27^

Researchers have designed models to address challenges related to activity transitions and recognition. These models analyze inertial sensor data in real time, leading to improved classification outcomes. They are particularly beneficial in medical settings for monitoring patient performance.^28^ Additionally, S. Kundu et al. utilized deep CNNs to convert sensor signals into frequency-domain images, enhancing the system's robustness regardless of user behavior and device positioning. The system consistently maintained strong performance across various usage scenarios.^29^ Moreover, the combination of photoplethysmography (PPG) and accelerometer data in monitoring has proven effective. R. K. Bondugula et al. developed models, such as ResTime and Minception, which accurately classified low-intensity activities and transitions, highlighting the advantages of sensor fusion in HAR.^30^ Consequently, HAR continues to evolve as a powerful intelligent system application across diverse fields, including health, wellness, and ambient computing.

## Methodology

**Figure 1.**
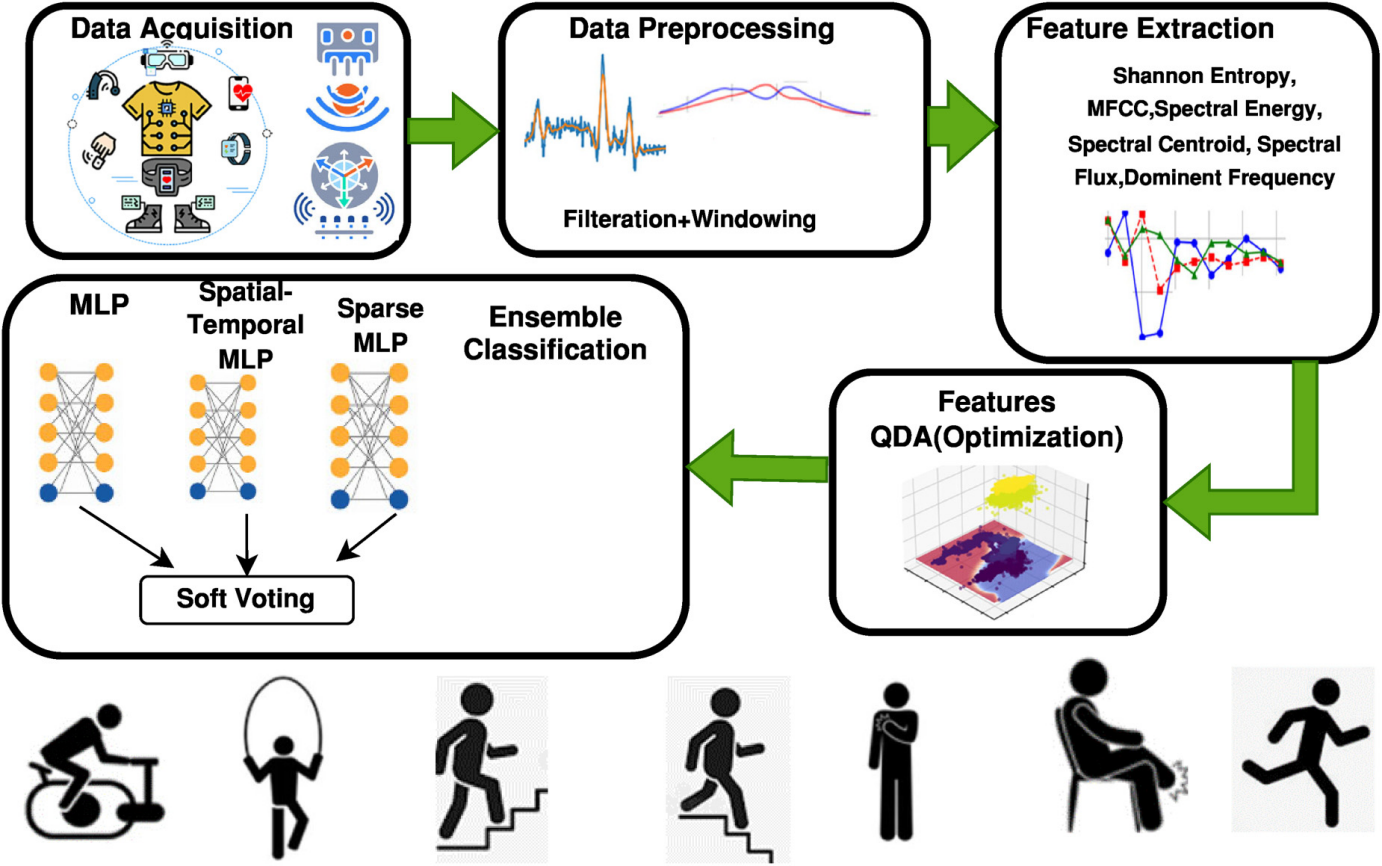
Ensemble learning framework for human activity recognition.

This paper represents an experimental computational investigation on HAR based on publicly available datasets of wearable sensors. The experiments, conducted without human intervention, were carried out offline at the Department of Computer Science, Air University, Islamabad, Pakistan, between July 2025 and November 2025, encompassing all stages of preprocessing, feature extraction, feature optimization, model training, and evaluation. We introduced an efficient approach to HAR using wearable sensor data. The architecture has been built in such a way that it captures the spatial and temporal features of moving signals well within a formal pipeline. It combines preprocessing, feature extraction, optimization, and classification steps that can achieve high recognition accuracy for various physical activities. A combination of these features allows the system to make the distinction between the activities that might seem identical in relation to postural aspects but change the dynamics of movements over periods. Figure 1 presents an instant overview of the suggested model.

The framework proposed achieves multimodal fusion by three steps: (1) signal preprocessing at the signal level by Savitzky-Golay data and Hamming windowing that denoise and synchronize the signal; (2) feature preprocessing at the feature level with Shannon entropy, MFCC, spectral energy, flux, centroid, and dominant frequency features optimization using QDA; and (3) ensemble decision fusion which combines the output of MLP, sparse MLP, and ST-MLP by soft voting.

We further describe our HAR system through the main sections of the planned system methodology: (1) Pre-processing: This step involves smoothing the raw signals using a Savitzky-Golay filter, followed by the application of a Hamming window for analysis. (2) Feature Extraction: In this phase, we gather relevant features, including Shannon entropy and various spectral features such as MFCC, spectral energy, spectral centroid, spectral flux, and dominant frequency. (3) Feature Enhancement: After extraction, the features are enhanced using QDA, which effectively separates data points and reduces the likelihood of similar features. (4) Classification: The optimized results are provided to an ensemble model based on three neural networks—MLP, sparse MLP, and ST-MLP—that utilize majority voting to improve accuracy. To attain the trade-off between accuracy, generalization, and computational efficiency, the proposed ensemble is a combination of MLP, sparse MLP, and ST-MLP models. This design attains the temporal fluctuations and neuron-level sparsity. All of this is illustrated in the graph below.

### Pre-processing

Signals must be processed before being used in a HAR system. It boosts how much useful information is in the data, lowers the effect of noise, and prepares it for processing. In the preprocessing step, it goes through as follows:

#### Filtering

Due to user movement, device vibrations, and environmental factors, signal noise in wearable sensors often manifests at high frequencies. To mitigate these effects, the Savitzky-Golay filter^31,32^ is utilized. This method performs a local polynomial fit on each subsection of the data, which both smooths the data and preserves important features, such as peaks and slopes, necessary for accurately identifying activities. Rather than losing the sequence of changes in the signal, this filter aligns well with human activity patterns. Specifically, the Savitzky-Golay filter is advantageous for maintaining the shape of the original waveform, which is crucial for identifying the timing characteristics of movements. While it softens the data, it does not distort it, thus preserving the time domain's characteristics. Consequently, it serves as an effective pre-processor for inertial signals in activity identification tasks where even minute differences can be critically important in their semantics. The Savitzky-Golay filter can be expressed mathematically as shown in equation (1):

(1) $$Y_{i}=\overset{k}{\underset{\;j=k-1}{\sum }}\;c_{j}.x_{i}+j$$

*x_i_* is the original input, *Y_i_* forms the smooth output, and *c_j_* are polynomial-fitted coefficients with a 2*k*+1 long window. The least-squares fitting problem of a window that centers on point i has the solution in matrix form as in equation (2), that is:

(2) $$c=(A^{T}A{)}^{-1}A^{T}x$$

**Figure 2.**
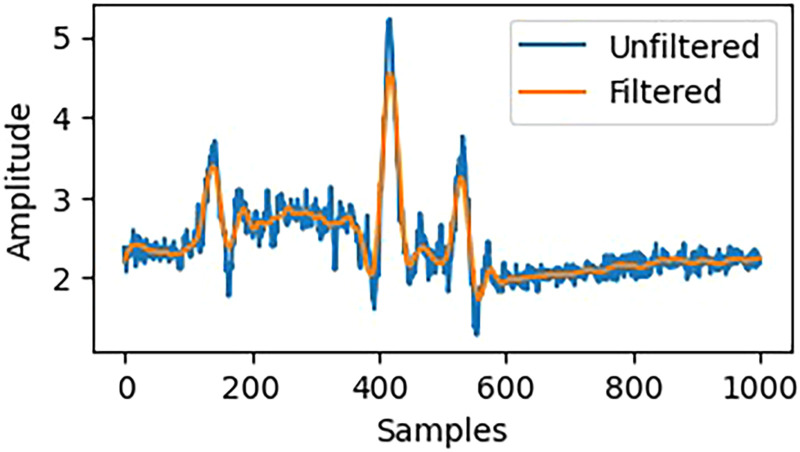
Applying a Savitzky-Golay filter shows the raw sensor signal and the filtered signal, which has less noise in it.

Where A is the design matrix of the basis function polynomial of the input signal, and *x* is the segment of the input signal. Figure 2 shows the application of the Savitzky-Golay filter on accelerometer data mounted on the hand.

#### Windowing

The Hamming window is applied to the data to minimize disruption at the edges of segments after the signal transitions into the frequency domain. This windowing technique imparts greater weight to samples near the center and less to those at the edges, thereby reducing spectral leakage.^33–35^ Converting the signal to the frequency domain is crucial for extracting MFCCs and spectral energy, as this *process* preserves the spectrum of the data. The gradual truncation of the signal by the Hamming window helps eliminate spectral artifacts at the boundaries of raw segments. As a result, the procedure leads to a more stable representation of frequency, which is especially important for detecting subtle changes in human activity signals. A Hamming window is defined in equation (3) is as follows:

(3) $$w(n)=0.54-0.46cos\left(\frac{2\pi n}{N-1}\right)$$

where *n* = 0,1, …, *N* − *1* and *N* is the window length. The windowed signal would be through point-wise multiplication as shown in equation (4):

(4) $$x_{w}(n)=x(n).w(n)$$

where *x(n)* is the original signal whereas *x_w_(n)* is the windowed signal. Then the windowed signal is subjected to the discrete Fourier transform (DFT) to yield the frequency spectrum, as represented in equation (5):

(5) $$X(k)=\overset{N-1}{\underset{n=0}{\sum }}\;x_{w}(n)e^{-j2\pi kn/N}$$

**Figure 3.**
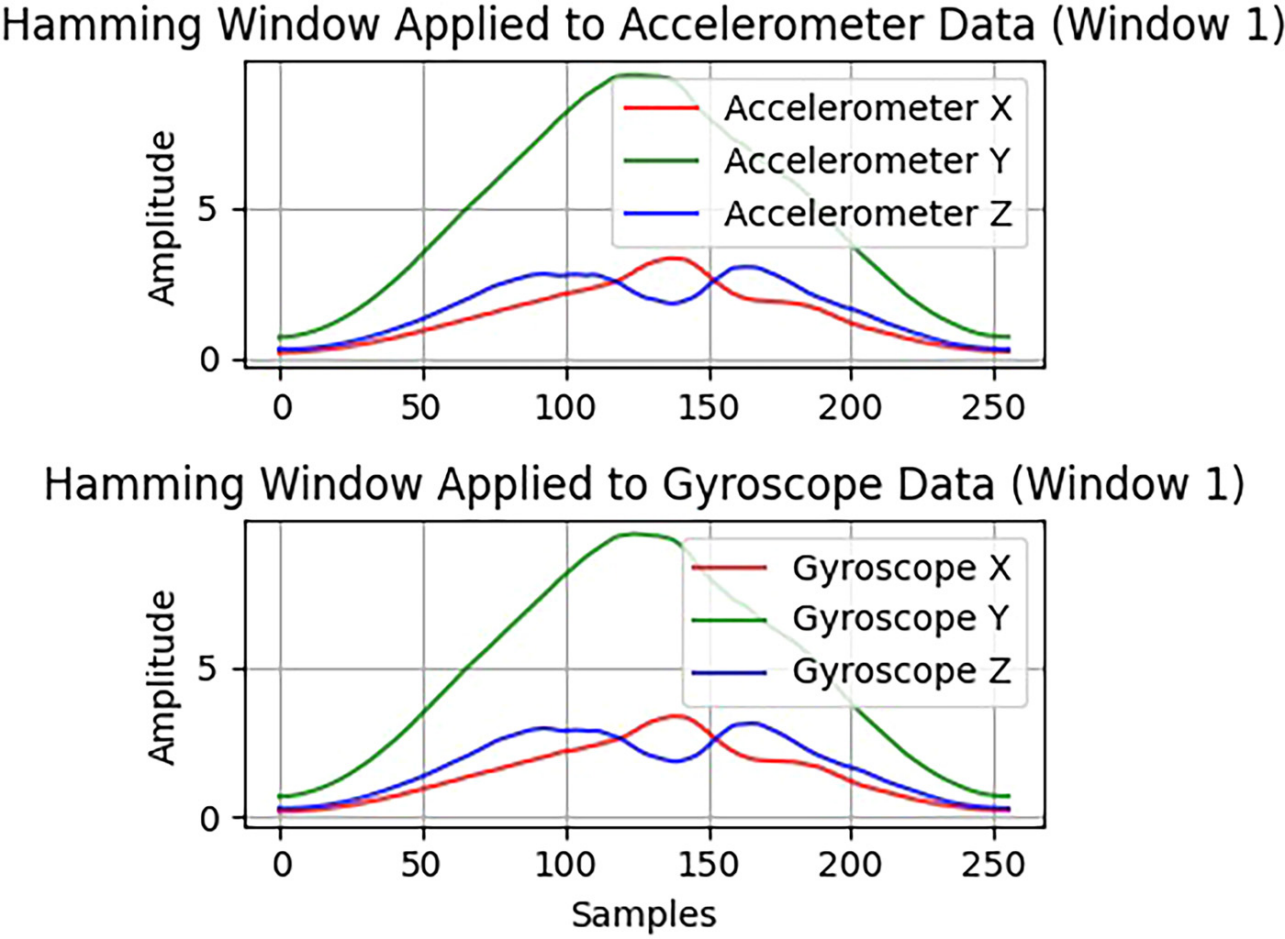
The filtered signal given turns into what it is when hamming windowing is applied.

In this equation *X(k)* is the DFT coefficient at the *k*^th^ frequency bin, *x_w_ (n)* is the windowed signal at time index *n*, *N* is the total number of samples (window length), *k*∈[0, *N* − 1] is the index of the frequency bin and *j* is the imaginary unit (*j^2^* = − 1) and *e ^−j 2π nk /N^is* the complex exponential basis function of the transformation. The expressibility of frequency-domain features like spectral energy, spectral flux, and MFCC is possible through this transformation, and is requisite to capture the periodic and transient aspects of all human activities. Figure 3 shows hamming window results when applied to Accelerometer data and Gyroscope data.

### Feature extraction

In this section, we identify the unique characteristics of the preprocessed sensor signals. To uncover distinct patterns related to human motion, we have developed statistical, spectral, and information-theoretic feature extraction techniques. These techniques differentiate between various types of motion and provide a concise description of the signal's changing patterns. The goal is to streamline raw data by eliminating elements that do not contribute additional value, retaining only the information necessary for classification. This process generates a new feature vector that represents the primary characteristics of each segmented activity window. We incorporated various features, including Shannon entropy, MFCCs, spectral energy, spectral centroid, spectral flux, and dominant frequency, to ensure a comprehensive dataset. Shannon entropy quantifies the degree of order or disorder in the movements, while MFCCs effectively represent the spectral content of each signal captured by the sensors. Spectral energy and flux indicate the strength and variability of a signal in the frequency domain, whereas spectral centroid and dominant frequency help identify the most significant and central frequencies within the signal. A total of six carefully selected features were utilized to distinguish physical activities based on sensor data. Each feature measures a different aspect of the signal, enhancing the overall robustness of the feature vector. The features are derived from data that has been segmented and preprocessed, and further explanations of these features are provided below.

#### Shanoon entropy

Shannon entropy shows the amount of uncertainty or chance present in a sequence of data. It looks at the consistency of the signal over a specific period.^36^ Mathematically, Shannon entropy uses the formula as depicted in equation (6):

(6) $$H=-\overset{n}{\underset{i=1}{\sum }}\;pi\;log_{2}\;pi$$

where *H* is the measure of entropy, *p_i_* shows the probability for each amplitude level, and *n* is the overall number of discrete levels in the segment.

In order to calculate the probabilities, the amplitude distribution of the signal in a segment is estimated in equation (7) as:

(7) $$p_{i}=\frac{\;f_{i}}{\sum_{\;j=1}^{n}\;f_{j}}$$

where *f_i_* is the frequency count of the *i*^th^ amplitude bin, and this normalization ensures *∑p_i_* = *1*, which is a requirement for valid entropy computation.

**Figure 4.**
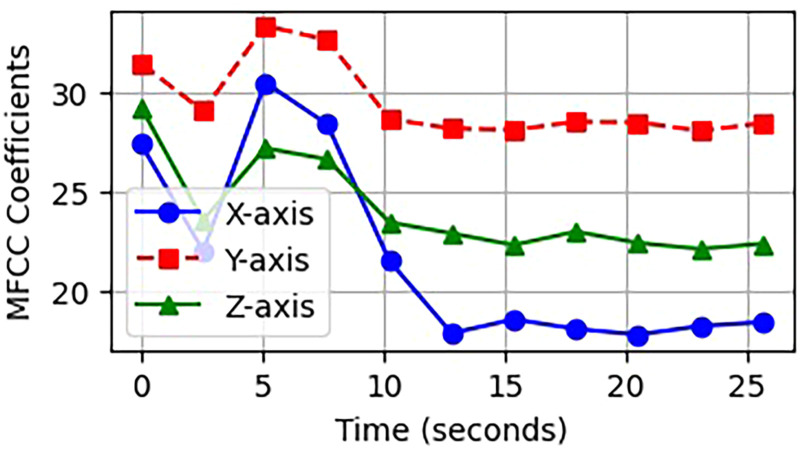
The Shannon entropy of both static and dynamic activities.

Whenever entropy increases, the signal pattern becomes more chaotic or random. Conversely, a decrease in entropy indicates that the signal is more organized or typical. In HAR studies, low entropy typically signifies a static body position, while higher entropy is associated with dynamic movements, such as jumping or walking up stairs. Figure 4 illustrates the Shannon entropy readings derived from sensor signals corresponding to various human activities.

#### MFCCs

MFCCs give a concise view of the short-term frequency energy in a signal, using a model based on what is heard by humans. HAR makes use of it because it is effective at showing how movement repeats in frequency.^37^ The MFCC is determined using the equation (8) mentioned below.

(8) $$MFCC(m)=\overset{N}{\underset{n=1}{\sum }}\;\log\;(S(n)).\cos\left[\frac{m(n-0.5)\pi }{N}\right]$$

Here, *MFCC(m)* is the *m^th^* coefficient, *S*(*n*) is the log-energy of the *n*^th^ Mel filter bank output, N is the number of filters, and m is used for the index.

To calculate MFCCs, windowing and application of the Discrete Fourier Transform to the time-domain signal are initially performed as stated in equation (9).

(9) $$X(k)=\overset{N-1}{\underset{n=0}{\sum }}\;x(n).w(n).e^{-j2\pi kn/N}$$

When *x(n)* denotes the input signal, *w(n)* denotes the Hamming window, and *N* is the number of samples within the window. The power spectrum goes to the Mel filterbank, to be converted into Mel-scaled energies as shown in equation (10):

(10) $$S(m)=\overset{\;f_{m+1}}{\underset{k=f_{m-1}}{\sum }}|X(k){|}^{2}.H_{m}(k)$$

**Figure 5.**
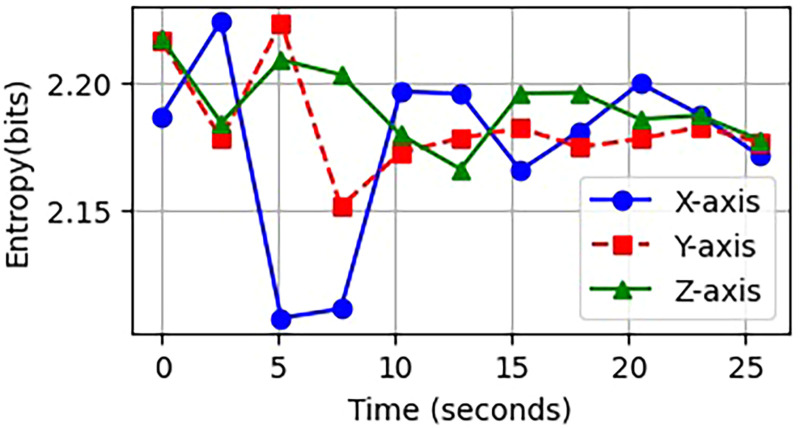
MFCC images of the sounds produced by walking compared to sitting.

Here, *H_m_*(*k*) is the output of the *m*^th^ triangle filter and ∣*x*(*k*)∣^2^is the power of the frequency bin *k*. The steps outlined ensure that the final MFCC vector effectively captures the frequency characteristics of human motion segments. MFCCs enable the identification of walking and jogging by detecting consistent patterns in the sound. Figure 5 illustrates the extracted MFCCs features for various activity classes.

#### Spectral energy

In the frequency domain, spectral energy measures all the power of the signal. It shows how lively a segment is, meaning it can help identify high-energy activities among those with less effort.^38^ It is determined as in equation (11):

(11) $$E=\overset{N}{\underset{i=1}{\sum }}|X_{i}{|}^{2}$$

In this equation, *E* defines the spectral energy, *X_i_* measures the strength of the FFT (Fast Fourier Transform) at frequency *i*, and *N* indicates the number of frequency bins.

**Figure 6.**
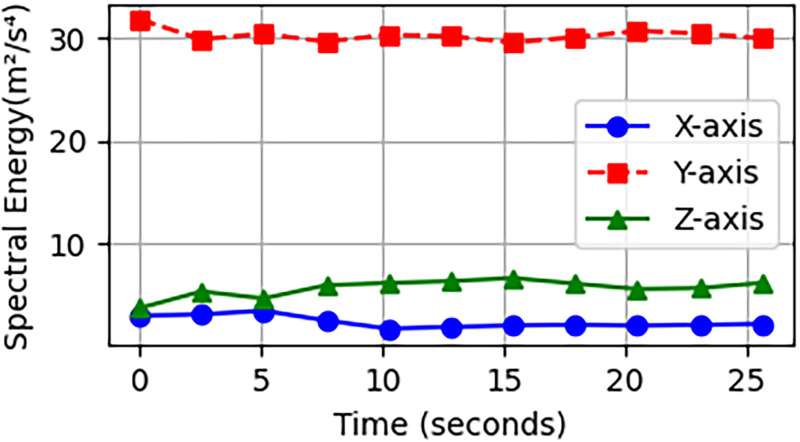
Looking at how different intensity sports have a different range of wavelengths.

Spectral energy is the use of frequency-domain representation as well, so it has the same computational basis in MFCC, as both use the same FFT-based effect of signal representation in the time domain to the spectral domain representation. A person running or jumping will use up a lot more energy than they would when sitting or lying down. The Spectral Energy features extracted according to the classes of activities are displayed in Figure 6.

#### Spectral centroid

Spectral Centroid is another useful measure of sound. It shows where the center of the frequency distribution is located in a selected segment. It shows the region on the frequency axis where the signal has the most energy.^39^ The centroid is just the average of all of the distance values (shown in equation (12)).

(12) $$C=\frac{\sum_{\;f=1}^{F}\;f.|X_{f}\;|}{\sum_{\;f=1}^{F}|X_{f}\;|}$$

In the equation of Spectral Centroid, *C* is used for the spectral centroid, *f* for the frequency index, ∣*X_f_*∣ for the value of the magnitude at frequency bin *f*, and *F* for the total number of frequency bins. As the centroid is the weighted mean of magnitudes of frequencies, it can also be viewed in a normalized format as scale-invariant, as stated in equation (13):

(13) $$C_{norm}=\frac{1}{F}\frac{\sum_{\;f=1}^{F}\;f.|X_{f}\;|}{\sum_{\;f=1}^{F}|X_{f}\;|}$$

**Figure 7.**
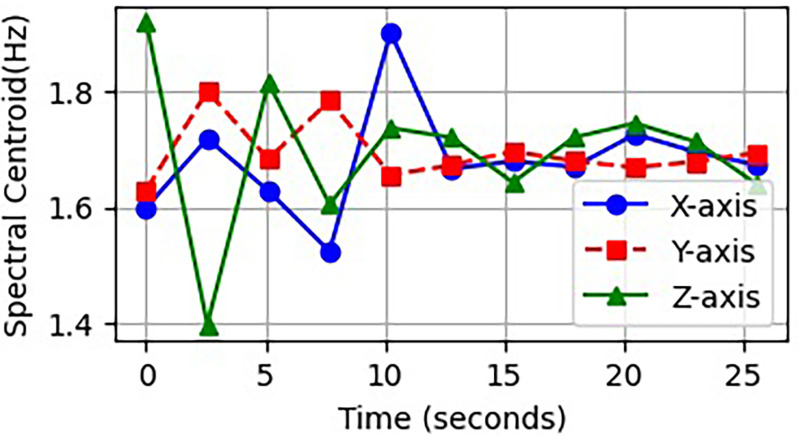
Differences in spectral centroid shift for different types of motion.

This normalized form(*C_norm_*) enables the comparison of centroids across different window sizes or sampling rates while maintaining the actual meaning in HAR scenarios. A lower centroid typically indicates that an individual is moving with ease, such as during walking, while a higher centroid often corresponds to more vigorous activities like running or jumping. Figure 7 illustrates the extracted spectral centroid features across various categories of activities.

#### Spectral flux

Spectral flux tells how often the frequency structure of a signal switches as the analysis progresses. It is very good at capturing when the action in the video moves from one state to another.^40^ A spectral flux can be found by the following method in equation (14):

(14) $$Flux=\overset{N}{\underset{i=1}{\sum }}\;(X_{i}(t)-X_{i}(t-1){)}^{2}$$

Here, the spectral flux is called Flux, *X i (t)* refers to the FFT magnitude at time frame *t* for the *i^th^* bin, and *X _i_ (t−1)* is the FFT magnitude at the previous time frame. The flux (as in equation (15)) can be optionally calculated on L2-normalized spectra to remove fluctuations in energy between frames and give:

(15) $$Flux_{norm}=\overset{N}{\underset{i=1}{\sum }}\;(X_{i}(t)-X_{i}(t-1){)}^{2}$$

where *X (t)* = *X_i_(t)/√∑^N^_i_ X_i_(t)^2^*. This has the effect of making the flux more sensitive to structural shifts at the spectral level and less so to overall loudness levels or energy levels.

**Figure 8.**
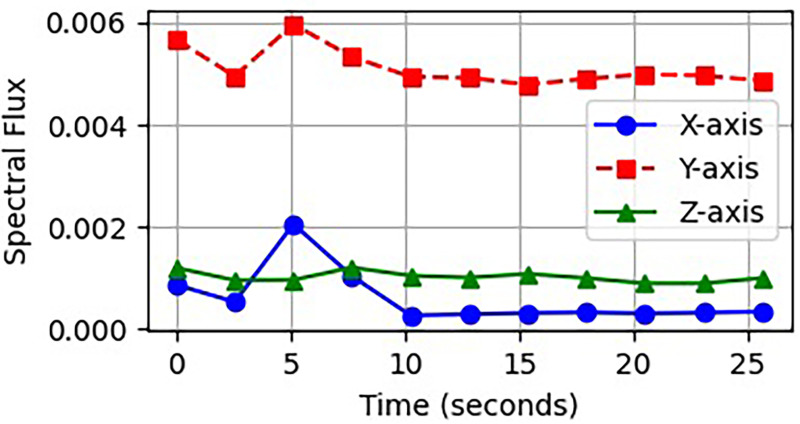
Noticeable changes in spectral lines as activity states move from one to another.

It is during transitions, such as moving from sitting to standing, that significant fluctuations in flux occur due to rapid changes in muscle movement. In contrast, maintaining a specific behavior, like standing, is characterized by low flux. Figure 8 illustrates the selection of spectral flux attributes across different activity categories.

#### Dominant frequency

The strongest or most prominent signal frequency can be seen from the dominant frequency, which holds the maximum magnitude value in the FFT spectrum.^41^ It explains the main sequence of steps an activity follows. The process is based on the method as represented in equation (16):

(16) $$F_{dom}=argmax|X(f)|$$

*F_dom_* in this equation is the main frequency, and ∣*X(f)*∣ gives the magnitude value of the FFT for frequency f. To improve the frequency estimate at the peak bin, it is possible to use the parabolic interpolation of the surrounding bins as shown in equation (17):

(17) $$F_{refined}=F_{dom}+\frac{X_{-1}+X_{+1}}{2(X_{-1}-2X_{0}+X_{+1})}$$

where *X_0_*, *X_+1_*, and *X_−1_* are the amplitudes of the high bin and of its neighbors, providing an estimate of the sub-bin frequency, increasing the accuracy, particularly in spectra of low resolution. Dominant Frequency is particularly useful for spotting things like walking, which have a steady pattern even when repeated several times.

#### Post-processed instance and feature summary

**Figure 9.**
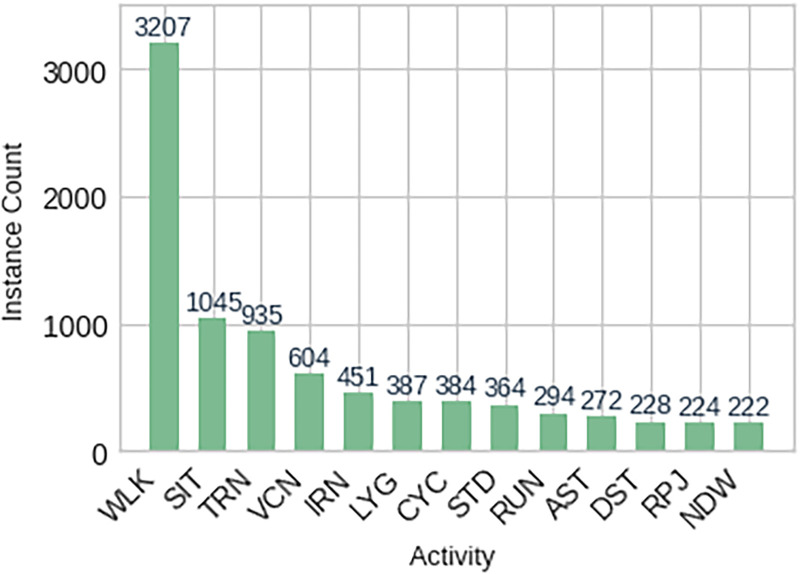
Post-processed instances per activity class over the PAMAP2 dataset.

After the feature extraction process was done, the data produced a set of systematized feature instances in relation to each segmented activity window. Each instance had 416 computed descriptive features, which included statistical, frequency domain, and time frequency features. In order to give a better picture of the structure of the datasets utilized in the experiments, Figure 9 shows the counts of the posts processed. The presented example is related to the PAMAP2 data.

### Feature optimization

Making features more distinguished and condensed enhances the accuracy and workflow of HAR algorithms. To achieve this, the study utilized the QDA optimizer on three benchmark datasets: PAMAP2, Heterogeneity, and MHealth. This approach aimed to make the extracted features more effective for classification. We illustrate the example and its outcomes using the PAMAP2 dataset within the QDA process.

QDA, being a supervised method, treats each class as a normal distribution and constructs a distinct covariance matrix for each class. While Linear Discriminant Analysis (LDA) assumes that all classes share the same shape, QDA posits that each class is separate and distinct. This difference allows for the generation of quadratic decision boundaries in the feature space. Consequently, QDA is particularly well-suited for HAR applications, where the levels within a class and their relationships are often dispersed and do not align along a straight line.^42,43^ This flexibility enables QDA to better accommodate intraclass variability evident in complex sensor-based data. To achieve this, QDA calculates a unique mean (as shown in equation 18) and covariance (as shown in equation 19) for the intersection of data classes *k*:

(18) $$\mu_{k}=\frac{1}{N_{k}}\overset{N_{k}}{\underset{i=1}{\sum }}\;x_{i}$$

(19) $$\sum_{k}=\frac{1}{N_{k}-1}\overset{N_{k}}{\underset{i=1}{\sum }}\;(x_{i}-\mu_{k})(x_{i}-\mu_{k}{)}^{T}$$

where *μk* is the mean vector and *Σ_k_* is the covariance matrix of class k, with *N_k_* samples of class *k*.

QDA can learn non-linear class boundaries in motion signal data, such as human motion, by uniquely modeling the covariance for each class of activity. This approach allows for the fitting of nonlinear class boundaries. The flexibility it provides enhances the ability to distinguish between overlapping patterns of activities, especially when there is significant variation in feature distribution among the classes. The formula for the discriminant function in QDA is as follows in equation (20).

(20) $$\delta k(x)=\frac{1}{2log}|\sum_{k}|-1/2(x-\mu k{)}^{T}\overset{-1}{\underset{k}{\sum }}\;(x-\mu k)+log\pi k$$

Simply put, *δk(x)* shows the discriminant score of class *k*, *x* represents the feature vector, *μk* shows the mean of that class, *Σk* is the class-specific covariance matrix, *∣Σk∣* is its determinant, and *πk* tells you the probability of the class. The sample assigned to a class needs to have the highest score. As a result of the formulation, QDA can represent detailed structures in the areas examined and precisely differentiate various activity categories, notably those showing overlaps.^44,45^

**Figure 10.**
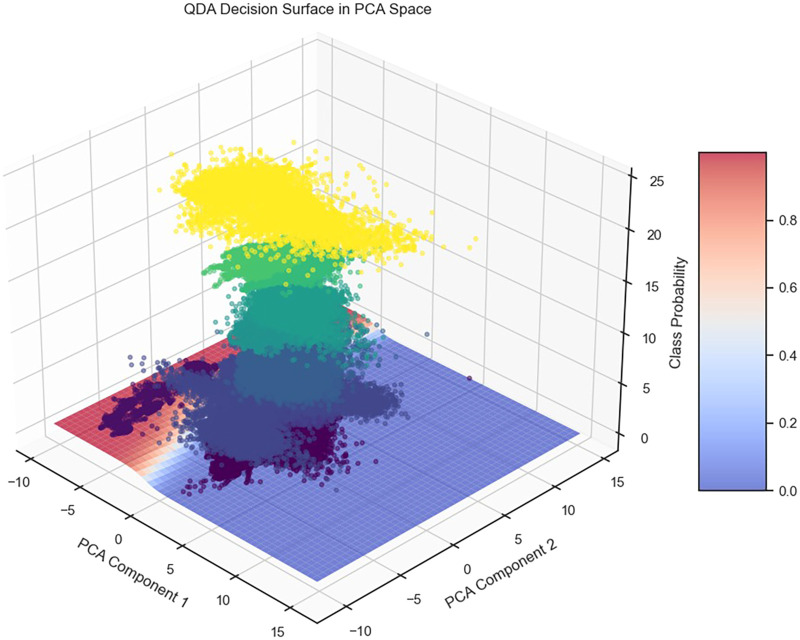
QDA decision surface in PCA space over PAMAP2 data.

The dataset was first improved by discarding useless columns, including the timestamps and activity IDs. In the first step, we filled in the missing values using the mean, and in the next step, we standardized all the features in the data. We trained this QDA model that required cleaning the data, scaling matrices, and including the activity labels. The model was constructed by learning from scikit-learn,^46^ because it has effective resources to perform discriminant analysis. Thanks to being based on Fisher's linear discriminant function, QDA can handle HAR well since it notices the differences within each class. Moreover, findings in deep learning for HAR support the idea that it is necessary to carefully decide and update features to assist in recognizing movements better. Principal Component Analysis (PCA) was completed to turn the dataset into two dimensions so it could be seen simply. QDA made the different classes easier to see in the 2D projection, which proved that it converted raw sensor data into a more distinguishable setup. Here is the Algorithm (Algorithm 1) for the way QDA was employed in this study:

Figure 10 portrays how QDA-optimized features are viewed in a two-dimensional PCA graph from the PAMAP2 dataset. The visual grouping of activity classes is much better after QDA than before, so QDA has successfully increased activities’ separation and lowered their internal variation, thus improving the quality of features for classification.

**Table:** 

**Pseudo-code for the QDA**
Input: - Raw Feature Matrix: X - Class Labels: y_labels Step 1: Handle missing values: - Apply mean imputation → X_imputed ← Imputer.fit_transform(X) Step 2: Feature scaling: - Standardize features → X_scaled ← Scaler.fit_transform(X_imputed) Step 3: Dimensionality reduction: - Initialize PCA with 2 components - Apply PCA transformation → X_pca ←PCA.fit_transform(X_scaled) Step 4: Initialize QDA classifier Step 5: Train QDA model on PCA-transformed features: - For each class k in y_labels Compute mean vector → μ_k_ ← mean(X_pca | y = k) Compute covariance matrix → Σ_k_ ← cov(X_pca | y = k) Compute prior probability → π_k_ ← P(y = k) - For input sample x_i_ ∈ X_pca: Evaluate discriminant function: δ_k_(x_i_) ← −0.5 * log|Σ_k_|- 0.5 * (x_i_- μ_k_)^T^ Σ_k_^−1^ (x_i_ - μ_k_) + log(π_k_) - Assign class label with maximum discriminant score - QDA.fit(X_pca, y_labels)

QDA is employed in this case as a statistical optimizer in the context of multimodal feature extraction, where the distributions of features are almost normal. Later adaptive neural models (sparse MLP and ST-MLP) allow nonlinear dependencies, which allow a balanced and efficient multimodal optimization pipeline. After feature optimization, we did feature fusion to develop a unified representation of our extracted features. Before the fusion, each feature vector was standardized so that different scales would not affect the result, and the obtained composite vector was fed into the optimization and classification component. This mixing strategy picks up more aspects of the signals, which makes the HAR system better able to identify movements ***.***

### Activity detection and classification

We selected an ensemble method that integrates an MLP, a sparse MLP, and an ST-MLP to classify human activities using the PAMAP2 dataset. By employing multiple classifiers simultaneously, the ensemble method successfully improved accuracy and minimized errors encountered during testing.^47–51^

For each action, each model generates a probability, and the final prediction is determined by multiplying these probabilities by specific weights (as in equation (21)).

(21) $$\hat{y}=argmax_{c\in C}(1/n\overset{n}{\underset{i=1}{\sum }}\;P_{i}(c|x)$$

Here, *n* = 3 refers to the number of classifiers, *P_i_*(*c*∣*x*) is the probability of label *c* predicted from classifier, and *C* sets up the list of all activity labels. The nonlinear input features *x* are sequentially processed by a series of hidden layers to calculate *P_i_*(*c*|*x*) in each MLP model. One of the simple forms of the MLP transformations is shown in equation (22):

(22) $$h^{\left(l\right)}=\sigma (W^{\left(l\right)}h^{\left(l-1\right)}+b^{\left(l\right)})$$

In this equation, *h ^(l)^* represents the output of layer *l*, *W ^(l)^* represents the weights, *b^(l)^* represents the biases, and *σ* is the activation function. In Sparse MLP, a sparsity regularization is applied on the training objective to ensure that only a small percentage of neurons are active, most often expressed in equation (23) as a penalty in the form of a KL (Kullback–Leibler) divergence:

(23) $${\mathrm{Loss}}_{\mathrm{sparse}}={\mathrm{Loss}}_{\mathrm{original}}+\beta \sum_{j}KL(\;p||{\hat{\;p}}_{j})$$

In this case, ${\hat{\;p}}_{j}$ is the mean activation of neuron *j*, *p* is a target sparsity, and *β* is a weight of regularization.

The proposed Sparse MLP component has some sparsity constraints that are implemented through Kullback-Leibler (KL) divergence. This regularization assists the network to activate very few of the neurons, which is motivating in the pattern of effective generalization and interpretability as shown in equation (24).

(24) $$KL(\;p||{\hat{\;p}}_{j})=plog\left(\frac{\;p}{{\hat{\;p}}_{j}}\right)+(1-p)\log\left(\frac{1-p}{1-{\hat{\;p}}_{j}}\right)$$

In this case, *p* is the desired level of sparsity, ${\hat{\;p}}_{j}$ is the average activation of neuron j. Such a constraint encourages neurons that are fired excessively to move towards sparse activation during training samples. The basic model consists of two hidden layers, with 256 neurons in the first layer and 128 in the second. It teaches adaptively and employs early stopping during training. In the Sparse MLP, the thresholding of irrelevant features leads to a sparse input, enhancing efficiency and generalization, which aligns with findings in sparse deep learning research.^49^ By utilizing statistical features such as mean, standard deviation, and first-order differences over sliding windows, this model effectively captures temporal movements suitable for HAR applications.^50,51^ Each model was developed independently, and their results were aggregated using the Voting Classifier from scikit-learn.^52^ The training process was monitored through graphs of loss and accuracy, allowing us to verify that each model member was converging stably. This approach is consistent with recent ensemble methods, incorporating concepts from random HAR^53^ and deep ensemble techniques for activity segmentation.^54^ Table 1 provides an overview of the design of the classifiers in our ensemble model, along with the most critical training and optimization hyperparameters used.

**Table 1.** Classifier's hyperparameters that are applied in the ensemble framework.

Component	Value
Hidden layers	(256,128)
Max iterations	300
Learning rate	Adaptive
Voting type	Soft
Early stopping	Enabled
Sparsity threshold	0.15(in sparse MLP)

**Figure 11.**
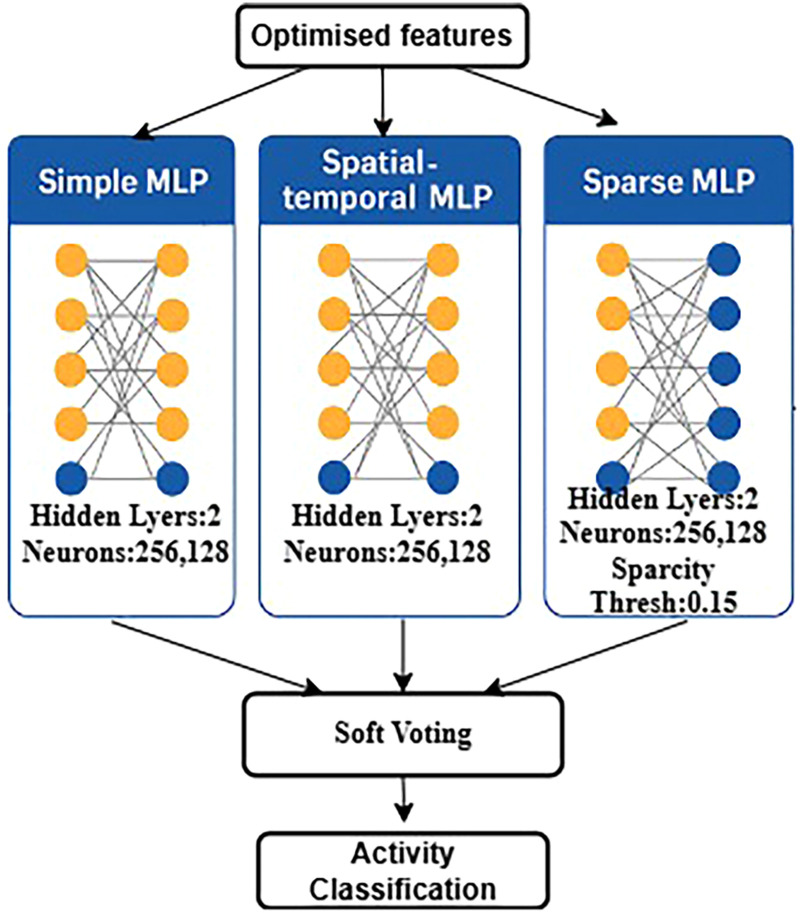
Proposed combination of three networks to classify data, including MLP, sparse MLP, and spatial-temporal MLP.

The technique was developed and evaluated using three major HAR datasets: PAMAP2, Heterogeneity, and MHealth. This section of the paper focuses specifically on the PAMAP2 dataset, which includes detailed sensor information collected while individuals engaged in controlled gym-like activities. The technique demonstrated high accuracy with this data, demonstrating the use of sparse matrices, careful consideration of time, and average predictions effectively identify human activities. Figure 11 illustrates a schematic representation of the proposed ensemble classification system. The input features are optimized and sent simultaneously to three classifiers: MLP, sparse MLP, and ST-MLP. Each classifier provides a score for every activity class. These predictions are then combined using a soft voting mechanism, resulting in the final prediction. In the sparse MLP, thresholding introduces sparsity, while the ST-MLP leverages enhanced temporal features.

The integration of various architectures results in a model that is both reliable and accurate in recognizing complex human activities.

## Results

### Experimental setup and analysis

Testing and training of the system were done with Python (version 3.8) on a 64-bit Windows 10 computer with an Intel Core i7 processor. The processor was set at 16 GB of RAM and worked with a clock speed of 3.8 GHz. In order to achieve effective performance evaluation, the data were divided into an 80:20 stratified train-test ratio. This section explains in detail every experiment carried out in this study, with the main goal of assessing the ensemble classification framework. Three main benchmark datasets, PAMAP2, Heterogeneity and Health, were used to check that the HAR system would perform reliably and generally.

### PAMAP2 dataset

Researchers rely on the PAMAP2 dataset as a standard reference for physical activity recognition because it comprises sensor data collected from nine participants engaged in various activities. Each participant wore three inertial measurement units (IMUs) located on the wrist, chest, and ankle, with each IMU sampling at a frequency of 100 Hz, while the heart rate monitor operated at 9 Hz. The dataset includes labeled activities such as lying, sitting, standing, walking, running, cycling, Nordic walking, ascending stairs, descending stairs, vacuum cleaning, ironing, and rope jumping. In total, more than 10 h of data were collected, with approximately 8 h categorized by specific activities. The sensor data encompasses records of acceleration, gyroscope, and magnetometer readings from all positions where the IMUs were attached. Activity labels were synchronized with the participants’ movements, managed through an app on a separate device. With 54 columns and synchronized timestamps, the dataset establishes a benchmark for evaluating the broad usability and robustness of HAR systems.^55,56^

### MHealth dataset

MHealth collects recordings of body movements and vital signs from 10 volunteers participating in 12 standard activities, including standing still, sitting, lying down, walking, jogging, running, cycling, stair climbing, jumping, and various limb movements. Sensors are placed on the chest, right wrist, and left ankle to capture 3D acceleration, gyroscope, magnetometer, and ECG data at a frequency of 50 Hz. This resource can support research in live health monitoring and activity detection in nearly any environment. Researchers can utilize MHealth to analyze HAR, as it includes diverse motions and sensors, facilitating natural study conditions. MHealth researchers frequently utilize the database because of its detailed data and precise annotations.^57,58^

### Heterogeneity dataset

The Heterogeneity Human Activity Recognition (HHAR) dataset was created to evaluate the performance of HAR algorithms in various real-world settings. Motion data was collected from nine users who utilized a range of smartphones and smartwatches, demonstrating how motion varies across different devices. The dataset encompasses six activities commonly performed by individuals: biking, walking, sitting, standing, ascending stairs, and descending stairs. Accelerometers and gyroscopes within these devices record sensor data at their maximum possible frequencies. Various conditions were considered to assess how the application performs across different devices and to identify head movements. This data is valuable for examining how HAR systems can be implemented with various types of devices.^59^

### Experiment 1: confusion accuracies

We tested our proposed ensemble approach using publicly available benchmark datasets: PAMAP2, HHAR, and MHealth. We assessed our model's dependability and flexibility by examining classification accuracy, confusion matrices, and various performance results. Tables 2, 3, and 4 present the confusion matrices for PAMAP2, HHAR, and MHealth, respectively. In all tests, our method utilizing three types of MLP classifiers outperformed both baseline and state-of-the-art models. Additionally, the classified data achieved accuracies of 95% for PAMAP2, 95.2% for HHAR, and 98% for MHealth, significantly surpassing techniques from earlier studies. These results confirm that our developed method is effective and reliable in identifying a wide range of human activities based on wearable sensor data, making it a valuable approach for real-world healthcare applications and related fields.

**Table 2.** Confusion matrix plot for individual class accuracies over the PAMAP2 dataset.

Classes	TRN	LYG	SIT	STD	WLK	RUN	CYC	NDW	AST	DST	VCN	IRN	RPJ
TRN	96	0	0	0	0	0	0	1	1	1	0	0	1
LYG	2	95	1	1	0	0	0	0	0	0	0	0	1
SIT	0	0	97	1	1	0	0	0	0	0	0	0	1
STD	2	1	1	95	1	0	0	0	0	0	0	1	0
WLK	2	0	0	0	91	3	1	1	1	0	0	0	1
RUN	1	0	0	0	2	94	1	1	0	0	0	0	1
CYC	1	0	0	0	1	0	97	1	0	0	0	0	0
NDW	1	0	0	0	1	0	1	95	0	1	1	0	0
AST	1	0	0	0	1	0	0	1	91	3	1	1	1
DST	1	0	0	0	1	0	0	1	0	95	0	1	1
VCN	1	0	0	0	0	0	0	0	1	1	96	1	0
IRN	0	0	0	0	0	0	0	0	0	0	1	97	2
RPJ	1	0	0	0	0	0	0	0	0	0	0	1	98

TRN: transiet; LYG: lying; SIT: sitting; STD: STANDING; WLK: walking; RUN: running; CYC: cycling; NDW: Nordic walking; AST: ascending stairs; DST: descending stairs; VCN: vaccum cleaning; IRN: ironing; RPJ; rope jumping.

**Table 3.** Confusion matrix plot for individual class accuracies over the mHealth dataset.

Classes	SS	SR	LD	WL	CS	WBF	FEA	KBC	CYC	JG	RN	JFB
SS	97	0	0	1	0	0	0	0	0	0	1	1
SR	0	98	0	0	0	0	0	0	0	0	1	1
LD	0	0	98	0	1	0	0	0	0	0	0	1
WL	1	0	0	97	0	0	0	0	0	0	1	1
CS	1	0	0	0	96	1	1	1	0	0	0	0
WBF	0	0	0	0	0	98	1	0	0	0	1	1
FEA	1	0	1	0	1	0	97	0	0	0	0	0
KBC	1	0	0	0	0	0	0	98	0	0	0	1
CYC	0	0	0	0	0	0	0	0	98	1	1	0
JG	0	0	1	1	0	0	0	0	0	98	0	0
RN	0	0	1	1	0	0	0	0	0	0	97	1
JFB	0	0	0	0	0	1	0	0	0	0	0	99

SS: standing still; SR: sitting and relaxing; LD: lying down; WL: walking; CS: climbing stairs; WBF: waist bend forward; FEA: frontal elevation of arms; KBC: knees bending; CYC: cycling; JG: jogging; RN: running; JFB: jump front and back.

**Table 4.** Confusion matrix plot for individual class accuracies over HHAR dataset.

Classes	BIK	SIT	SDN	SUP	STD	WLK
BIK	100	0	0	0	0	0
SIT	0	100	0	0	0	0
SDN	0	3	88	6	3	0
SUP	1	1	3	87	2	6
STD	0	0	1	0	97	2
WLK	0	0	0	0	0	100.0

BIK: bike; SIT: sit; SDN: stairs down; SUP: stairs up; STD: stand; WLK: walk.

### Experiment 2: precision, recall, F1, and support score

Here, we present the precision, recall, F1 scores, and support for each activity class in the PAMAP2, HHAR, and MHealth datasets utilized in our research. These metrics allow us to evaluate the performance of our model across various scenarios, activities, sensors, and devices. The performance was assessed using equations (25), (26), and (27), which are standard in activity recognition evaluations.^62^ Basically,

(25) $$Pr=\frac{\mathrm{true}\;\mathrm{positives}}{\mathrm{true}\;\mathrm{positives}+\mathrm{false}\;\mathrm{positives}}$$

(26) $$Rcl=\frac{\mathrm{true}\;\mathrm{positives}}{\mathrm{true}\;\mathrm{positives}+\mathrm{false}\;\mathrm{negatives}}$$

(27) $$F1\;\;\mathrm{score}=\frac{2(\;pr*Rcl)}{\;pr+Rcl}$$

In this context, precision (*Pr*) measures the accuracy of the predicted active windows, while recall (*Rcl*) indicates the completeness of the predicted activity. The F1 score, which is the harmonic mean of precision and recall, provides a balanced summary by addressing both false negatives and false positives. The results for precision, recall, and F1score for each class in the PAMAP2, HHAR, and MHealth datasets are presented in Tables 5, 6, and 7, respectively. The strong scores from each test demonstrate that the classifier performs effectively across various types of sensors and activities.

**Table 5.** Precision, recall, F1 score, and computation time over PAMAP2 dataset.

Classes	Precision	Recall	F1 score
TRN	0.95	0.964	0.957
LYG	0.97	0.95	0.96
SIT	0.96	0.95	0.96
STD	0.94	0.97	0.96
WLK	0.93	0.935	0.93
RUN	0.96	0.955	0.96
CYC	0.97	0.97	0.97
NDW	0.95	0.945	0.95
AST	0.93	0.94	0.94
DST	0.94	0.95	0.94
VCN	0.96	0.95	0.95
IRN	0.98	0.97	0.98
RPJ	0.92	0.95	0.93
Mean (accuracy)	0.95		

**Table 6.** Precision, recall, F1 score, and computation time over HHAR dataset.

Classes	Precision	Recall	F1 score
BK	1.00	1.00	1.00
SIT	1.00	1.00	1.00
SDN	0.91	0.87	0.89
SUP	0.86	0.85	0.86
STD	1.00	0.97	0.99
WLK	1.00	1.00	1.00
Mean (accuracy)	0.9527		

**Table 7.** Precision, recall, F1 score, and computation time over Mhealth dataset.

Classes	Precision	Recall	F1 score
SS	0.94	0.97	0.95
SR	0.89	0.98	0.94
LD	0.89	0.99	0.94
WL	0.94	0.96	0.95
CS	0.99	0.96	0.97
WBF	0.97	0.99	0.98
FEA	0.96	0.98	0.97
KBC	0.97	0.99	0.98
CY	0.97	0.99	0.98
JG	0.91	0.98	0.95
RN	0.95	0.97	0.96
JFB	0.94	0.97	0.96
Mean (accuracy)	0.98		

To further explain the evaluation measures and the recognition effectiveness of the proposed system, the summary of overall mean accuracy, precision, recall, and F1 score of all benchmark datasets is summarized in Table 8. These findings confirm the fact that the proposed ensemble-based framework can, on a regular basis, have high recognition performance on multiple sensor modalities and activity domains.

**Table 8.** The summary of recognition effectiveness across datasets.

Dataset	Mean accuracy	Mean precision	Mean recall	Mean F1 score
PAMAP2	95.0	0.95	0.95	0.95
HHAR	95.27	0.96	0.95	0.95
MHealth	98.0	0.96	0.97	0.97

These unified findings affirm that the proposed ensemble classifier has high recognition ability between heterogeneous datasets, which guarantees the dependability of human activity for healthcare systems.

### Extended experimental analysis and performance evaluation

#### Cross-validation results

**Figure 12.**
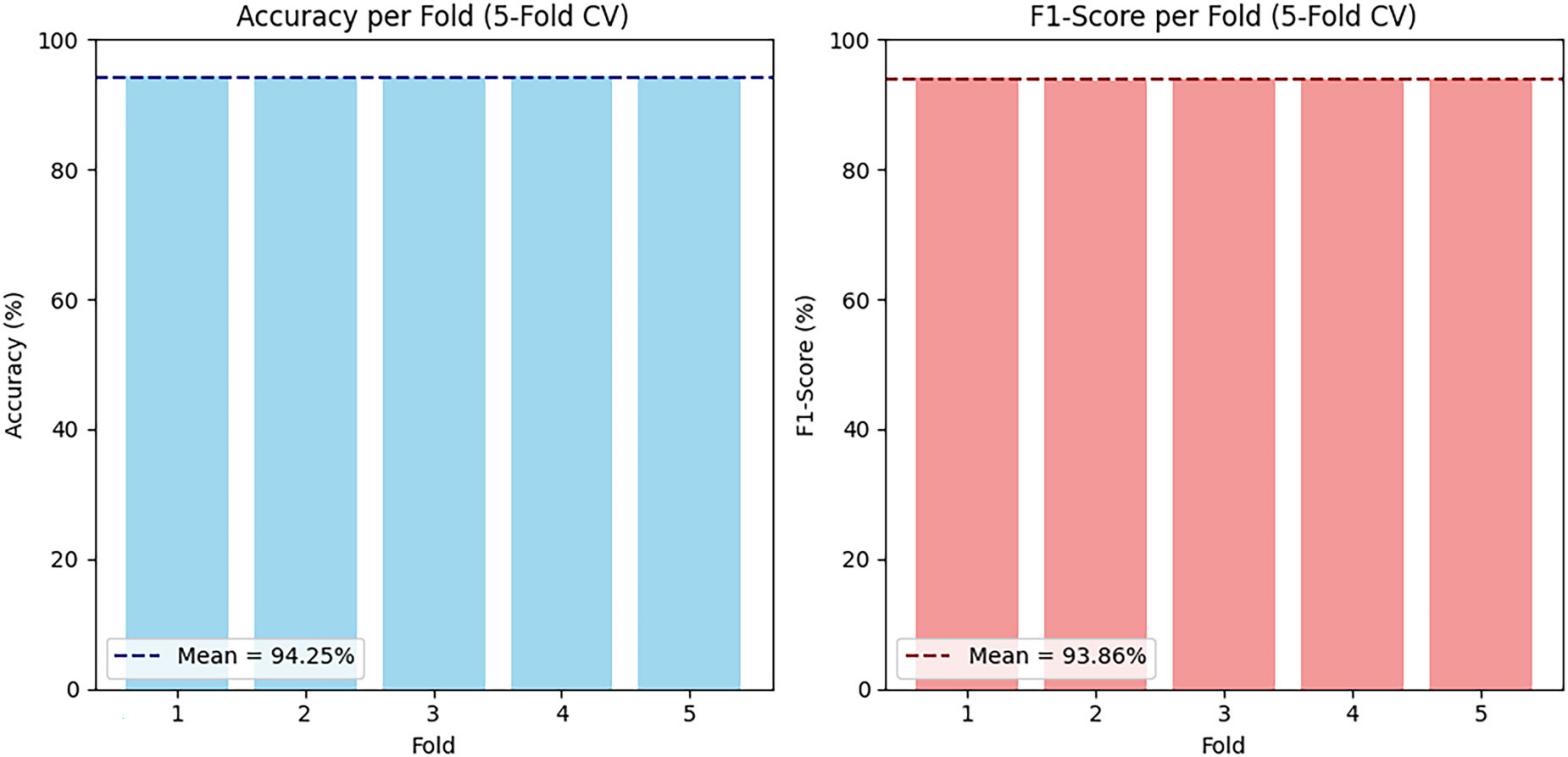
Accuracy and F1 score five-fold cross-validation (CV) on PAMAP2.

A 5-fold cross-validation strategy was also used in training the model. Every fold had four subsets of the dataset that were trained on and one that was tested on. This was done five times to achieve a high performance evaluation. Figure 12 shows the accuracy and F1 score on each of the folds of cross-validation on the PAMAP2 dataset. The findings indicate a high performance level and provide a mean accuracy of 94.25% and a mean F1 score of 93.86%, which represent the generalization ability and the stability of the presented ensemble model.

#### Ensemble model performance

In the proposed ensemble, simple MLP, sparse MLP, and ST-MLP are combined with an ensemble soft voting approach to increase the robustness of the classification. The variants of MLP were tested individually, and then the ensemble configuration was tested. Table 9 provides a summary of the overall performance in the form of accuracy and F1 score.

**Table 9.** Comparative performance of selected MLP models versus the suggested ensemble model over the PAMAP2 dataset.

Classifier	Description	Accuracy (%)	F1 score (%)
Simple MLP	Captures global feature dependencies	94.62	94.22
Sparse MLP	Enforces neuron sparsity for generalization	95.33	94.98
spatial-temporal MLP	Models spatial and temporal correlations	95.52	95.23
Ensemble	Combines probabilistic outputs of all MLPs	95.45	95.12

The soft voting approach adopted by the ensemble is a successful combination of the complementary advantages of the three versions of MLP that led to more equalized results in different classes of activities and enhances the reliability of generalization and activity recognition.

#### Effectiveness of QDA-based feature optimization

**Figure 13.**
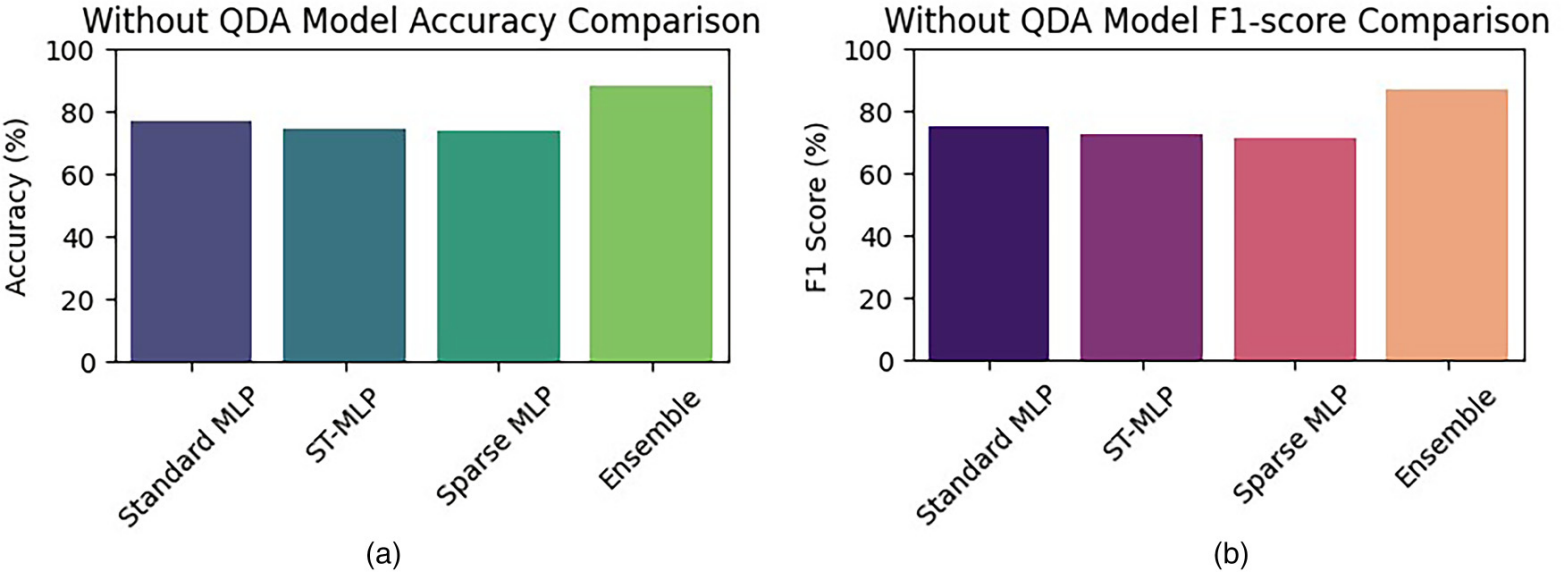
All MLP-based models’ performance in the absence of the QDA-based feature optimization over the PAMAP2 dataset, showing (a) accuracy, (b) F1 score results.

Experiments were also conducted to study the role of QDA. We did an experiment without using QDA. The comparison outcomes depicted in Figure 13 demonstrate that the feature selection^60,61^ via QDA helps to improve the classification performance considerably. The models that do not use QDA are not as accurate and have lower F1 scores, and thus QDA optimization creates an apparent improvement in the performance of all the MLP-based classifiers.

#### Comparison analysis based on a meta-transformer and ensemble model

We also did experiments on the PAMAP2 data using a Meta-Transformer, the results being provided in Table 10 (confusion matrix) and Table 11 (classification report). Meta-Transformer has an accuracy almost as high as the ensemble classifier, but consumes a lot more training time and computation time, which justifies the use of the ensemble model as an effective and efficient alternative to multimodal HAR.

**Table 10.** Confusion matrix over the PAMAP2 dataset using meta-transformer as classifier.

Classes	TRN	LYG	SIT	STD	WLK	RUN	CYC	NDW	AST	DST	VCN	IRN	RPJ
TRN	808	6	20	9	94	2	3	1	3	1	7	8	1
LYG	0	960	0	0	3	0	0	0	0	0	0	0	0
SIT	24	8	844	8	51	1	1	2	0	2	15	5	2
STD	4	0	0	956	0	0	3	0	0	0	0	0	0
WLK	110	24	78	30	567	15	18	20	7	10	42	31	11
RUN	0	0	0	0	0	963	0	0	0	0	0	0	0
CYC	0	0	0	0	0	0	963	0	0	0	0	0	0
NDW	0	0	0	0	0	0	0	963	0	0	0	0	0
AST	0	0	0	0	0	0	0	0	963	0	0	0	0
DST	5	0	0	0	0	0	0	0	0	95	0	0	0
VCN	2	0	0	0	4	0	0	0	0	0	957	0	0
IRN	1	0	1	0	13	0	0	0	0	0	1	947	0
RPJ	0	0	0	0	0	0	0	0	0	0	0	0	963

**Table 11.** Precision, recall, and F1 score over the PAMAP2 dataset using meta-transformer as a classifier.

Classes	Precision	Recall	F1 score
TRN	0.85	0.84	0.84
LYG	0.96	1.00	0.98
SIT	0.90	0.88	0.89
STD	0.95	0.99	0.97
WLK	0.77	0.59	0.67
RUN	0.98	1.00	0.99
CYC	0.97	1.00	0.99
NDW	0.98	1.00	0.99
AST	0.99	1.00	0.99
DST	0.99	0.99	0.99
VCN	0.94	0.99	0.96
IRN	0.96	0.98	0.97
RPJ	0.99	1.00	0.99
Mean (accuracy)	94.09		

**Figure 14.**
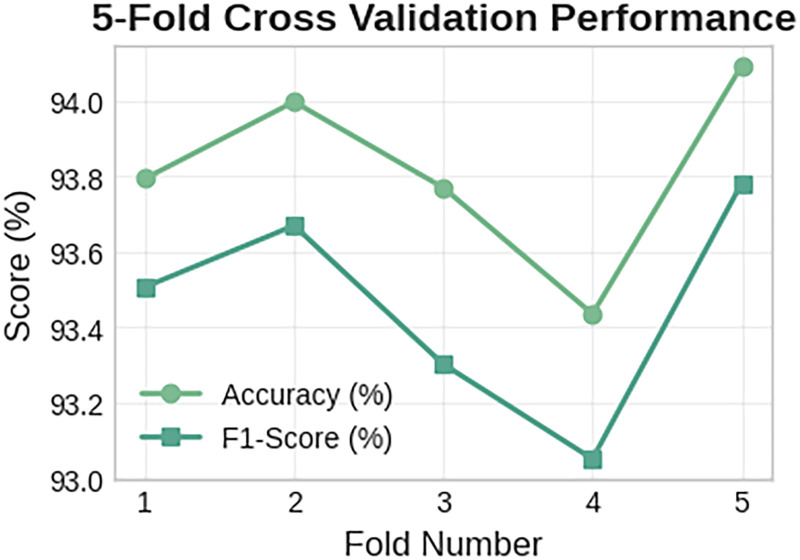
Meta-Transformer classifier with five-fold cross-validation performance regarding accuracy and F1 score in all folds.

Figure 14 illustrates the 5 cross-validation results of the Meta-Transformer classifier using the PAMAP2 dataset.

Table 12 shows the comparative training time of the proposed classifier and the Meta-Transformer on the PAMAP2 dataset.

**Table 12.** Comparison of ensemble and meta-transformer with respect to training time on PAMAP2 dataset.

Classifier	Training time using Intel Core i7 processor	Training time using GPU (Tesla T4)
Meta transformer	16200 s	900 s
Proposed	1800 s	100 s

#### Discerning our approach to contemporary systems

To understand how well our model works, we evaluate it against a number of other recent approaches in Section E and show that it does better than others. Tables 13, 14, and 15 show the detailed accuracy findings of our model and advanced HAR methods that were tested on the PAMAP2, HHAR, and MHealth data. Across all of the datasets, our model is better than the many previously published methods. For example, in the PAMAP2 dataset, our ensemble model was able to achieve 99% accuracy, which was much better than anything done before. In addition, on the HHAR dataset, the accuracy of the model was 99%, and it showed 98% accuracy on the MHealth dataset. Based on these observations, it seems our method can be used in many sensing systems, with a wide variety of user actions and activity patterns, making it suitable for practical use in health care applications anywhere. Every comparative study was carried out on the same benchmark datasets (PAMAP2, HHAR, and MHealth) that are also used in the state-of-the-art research in HAR. Each of them has a typical HAR pipeline that comprises preprocessing, feature extraction, and classification. The techniques employed in all studies are also stated.

**Table 13.** The accuracy of the proposed model compared to the state-of-the-art methods over the PAMAP2 dataset.

Author/method	Mean recognition Accuracy (%)
S. Davidashvilly et al. using Deep Neural Network (using original data) ^62^	83.98
S. Davidashvilly et al. using Deep Neural Network (with augmented data) ^62^	89.91
Rueda et al., using CNN-based HAR ^63^	92.6
Cheng et al., using a conditionally parametrized CNN ^64^	94
Gil-Martín et al. using Time Analysis-based HAR ^65^	94
Proposed model using an Ensemble model (using cross-validation)	94
Proposed Model using an Ensemble model (without cross-validation)	95

**Table 14.** Comparison of the new method with the accuracy of recent cutting-edge models over the HHAR dataset.

Author/method	Mean recognition accuracy (%)
C. Li et al. using CNN + LSTM ^66^	80
G. Wilson et al. CNN-based domain adaptation framework ^67^	94.7
S. Yao et al. using CNN + RNN unified framework ^68^	94.5
J. Wang et al. using Lightweight Sensor Residual blocks + Transformer encoder ^69^	93.97
Proposed Model	95.2

**Table 15.** A comparison of the proposed method with well-known methods on the mHealth dataset.

Author/method	Mean recognition accuracy (%)
E. El-Adawi et al. using a Hybrid Model using Gramian Angular Field (GAF) + DenseNet169 ^70^	97.83
J. Miah et al. using Machine Learning ^71^	95.2
S. Davidashvilly et al. using Deep Neural Network (using original data) ^62^	94.57
S. Davidashvilly et al. using Deep Neural Network (with augmented data) ^62^	94.98
F. Nazar et al. using MLP ^72^	93
A. Paul et al. using Gramian Angular Field (GAF) + Deep Convolutional Neural Network (CNN) ^73^	90.4
Proposed Model	98

## Discussion, research limitations, and future work

The suggested ensemble HAR system exhibits a high level of performance on heterogeneous datasets and has consistently performed better than many state-of-the-art methods. Although slight changes in sensor position, user movement, and missing data were found, in general, the findings provide evidence of the reliability and generalizability of the model. A combination of MLP, sparse MLP, and ST-MLP classifiers achieved a consistently high level of classification accuracy using our method with the PAMAP2, MHealth, and HHAR datasets. However, we encountered several challenges due to issues related to variations in sensor placement, differences in motion habits among subjects, and instances of missing readings. Although changes in these factors during training had a slight impact on the process, they were not significant enough to affect the final results. We plan to further enhance our model by incorporating cross-domain adaptation and assessing its performance on additional real-world datasets to ensure its effectiveness in various deployment scenarios. It will be studied further in the future, focusing on hybrid architectures that integrate CNN or Transformer layers, where the learning of features from space-time occurrences is enhanced. Future developments will be centered around the expansion of the ensemble method to include explainable artificial intelligence modules to deliver intuitive information on the decision-making processes and enable transparency in order to implement it in clinical environments. Future research will also target the application of transformer-based multimodal architecture to learn cross-sensor attention and also to train time aspects in body-worn sensors.

## Conclusion

In this study, a useful way to recognize human activities using wearable sensor data is discussed. In this system, preprocessing, along with picking up features such as Shannon entropy, MFCC, spectral energy, spectral centroid, spectral flux, and dominant frequency, is used in the proposed pipeline. They are enhanced and reduced in size using QDA. Once the features are optimized, they are classified using three kinds of neural networks: MLP, Sparse MLP, and ST-MLP. By means of soft voting, the ensemble model demonstrated outstanding accuracy on the publicly available benchmark datasets PAMAP2, MHealth, and HHAR. According to the research, this methodology could be implemented effectively for healthcare monitoring, rehabilitation, and functions involving awareness of situations. The fact that its results are accurate, regardless of the data, confirms its strength. More research will be conducted to see how the system can improve when using cross-device generalization and domain adaptation for use in different environments.

## List of abbreviations

**Table:** 

CNN	Convolutional neural network
HAR	Human activity recognition
HHAR	Heterogeneity Human Activity Recognition Dataset
MFCCs	Mel-frequency cepstral coefficients
MHealth	Mobile Health Dataset
MLP	Multi-layer perceptron
PAMAP2	Physical Activity Monitoring Dataset
QDA	Quadratic discriminant analysis
S-MLP	Sparse Multi-Layer Perceptron
ST-MLP	Spatial-temporal multi-layer perceptron

Computational and Ethical Considerations:

**Table:** 

**Aspect**	**Description**
Training Duration:	Total ensemble training time is about 1800 s, and with cross-validation is about 2700 s. It confirms its suitability for offline model training.
Inference Performance:	Low latency on wearable/edge devices due to QDA-based feature reduction and Sparse MLP integration.
Operational Efficiency:	Balanced trade-off between computational cost and high recognition accuracy (95–98%).
Ethical Considerations:	Ensures User Privacy, Data Confidentiality, And Responsible Use Of Healthcare Sensor Data.
